# Activation of COUP-TFI by a Novel Diindolylmethane Derivative

**DOI:** 10.3390/cells8030220

**Published:** 2019-03-07

**Authors:** Kyungsil Yoon, Chien-Cheng Chen, Asuka A. Orr, Patricia N. Barreto, Phanourios Tamamis, Stephen Safe

**Affiliations:** 1 Institute of Biosciences and Technology, Texas A&M Health Science Center, Houston, TX 77030, USA; 2 Division of Translational Science, National Cancer Center, Goyang-si, Gyeonggi-do 10408, Korea; 3 Department of Veterinary Physiology and Pharmacology, Texas A&M University, College Station, TX 77843, USA; ccchen112@gmail.com; 4 Department of Chemical Engineering, Texas A&M University, College Station, TX 77843, USA; asukaorr@tamu.edu (A.A.O.); patricia.barreto@tamu.edu (P.N.B.)

**Keywords:** COUP-TFI, Egr-1, activation, DIM-C-Pyr-4, Sp proteins

## Abstract

Chicken ovalbumin upstream promoter-transcription factor I (COUP-TFI) is an orphan receptor and member of the nuclear receptor superfamily. Among a series of methylene substituted diindolylmethanes (C-DIMs) containing substituted phenyl and heteroaromatic groups, we identified 1,1-bis(3′-indolyl)-1-(4-pyridyl)-methane (DIM-C-Pyr-4) as an activator of COUP-TFI. Structure activity studies with structurally diverse heteroaromatic C-DIMs showed that the pyridyl substituted compound was active and the 4-pyridyl substituent was more potent than the 2- or 3-pyridyl analogs in transactivation assays in breast cancer cells. The DIM-C-Pyr-4 activated chimeric GAL4-COUP-TFI constructs containing full length, C- or N-terminal deletions, and transactivation was inhibited by phosphatidylinositol-3-kinase and protein kinase A inhibitors. However, DIM-C-Pyr-4 also induced transactivation and interactions of COUP-TFI and steroid receptor coactivators-1 and -2 in mammalian two-hybrid assays, and ligand-induced interactions of the C-terminal region of COUP-TFI were not affected by kinase inhibitors. We also showed that DIM-C-Pyr-4 activated COUP-TFI-dependent early growth response 1 (Egr-1) expression and this response primarily involved COUP-TFI interactions with Sp3 and to a lesser extent Sp1 bound to the proximal region of the Egr-1 promoter. Modeling studies showed interactions of DIM-C-Pyr-4 within the ligand binding domain of COUP-TFI. This report is the first to identify a COUP-TFI agonist and demonstrate activation of COUP-TFI-dependent Egr-1 expression.

## 1. Introduction

Chicken ovalbumin upstream promoter-transcription factor I (COUP-TFI) is an orphan nuclear receptor and was initially identified as a transcription factor important for expression of the chicken ovalbumin gene [1,2,3]. Chicken ovalbumin upstream promoter-transcription factor II (COUP-TFII) [4], the second member of this sub-family, is highly homologous to COUP-TFI and both proteins exhibit the characteristic domain structure of other nuclear receptors; this includes the N- and C-terminal A/B and E/F domains, a hinge region (C), and a DNA binding domain (D) [5,6,7]. Chicken ovalbumin upstream promoter-transcription factors function in the absence of exogenous ligand and exhibit both transcriptional activation and repression activity. Chicken ovalbumin upstream promoter-transcription factors can influence transcription by binding as homodimers to direct repeat elements (AGGTCA) with various spacers and their interaction with other DNA bound receptor complexes forms the basis for some of their effects on transcription [8,9]. For example, the binding of COUP-TFI to direct repeat (DR) elements and palindromic response elements that are cognate DNA binding sequences for other nuclear hormone and orphan receptors can inhibit their hormone-induced and/or basal activity by displacing or disrupting the DNA bound-receptor complex. There are reports showing that COUP-TFs inhibit estrogen receptor, retinoic acid receptor, retinoic X receptor, peroxisome proliferator-activated receptor (PPAR), and hepatocyte nuclear factor 4-mediated gene expression [8,10,11,12,13,14,15,16,17,18,19,20,21,22,23,24]. 

Chicken ovalbumin upstream promoter-transcription factor I also modulates gene expression through interaction with other proteins including nuclear receptor corepressors and coactivators [25,26,27,28,29] and through cooperative interactions with other transcription factors including specificity proteins (Sps) [27,30,31,32,33,34]. Interactions of COUP-TFI with DNA-bound Sp1 and Sp3 and cooperative interactions of both COUP-TFI and Sp1/Sp3 with transcription factor II B (TFIIB) and other proteins are important for silencing the human luteinizing hormone receptor gene expression [34]. COUP-TF1 activates non-steroidal anti-inflammatory drug-activated gene 1 (NAG-1) in colon cancer cells through interactions with Sp proteins bound to proximal GC-rich sites in the NAG-1 promoter. Moreover, activation of the early growth response-1 (Egr-1) gene by COUP-TF has also been linked to interactions of COUP-TFI with Sp1 protein bound to proximal GC-rich motifs at 64 to 46 in the Egr-1 promoter [27]. 

Research in this laboratory has identified a series of methylene-substituted diindolylmethanes (C-DIM) analogs, namely 1,1-bis(3′-indolyl)-1-(p-substitutedphenyl)methanes which activate nuclear orphan receptors PPARγ and nerve growth factor-induced Bα (NGF-I-Bα, Nur77) [35,36,37,38]. We have also prepared a series of 1,1-bis(3′-indolyl)-1-(heteroaromatic)methane analogs (hetero-aromatic C-DIMs) as possible agonists for COUP-TFI. 1,1-Bis(3′-indolyl)-1-(4-pyridyl) methane (DIM-C-Pyr-4) has been identified as a unique compound among as series of heteroaromatic C-DIMs that activates a COUP-TFI-GAL4 chimera containing COUP-TFI fused to the DBD of GAL4 in MCF-7 and ZR-75 breast cancer cells transfected with a construct (GAL4-luc) containing 5-tandem GAL4 response elements linked to luciferase. DIM-C-Pyr-4 activates both the N- and C-terminal domains of COUP-TFI and these responses were differentially inhibited by kinase inhibitors. DIM-C-Pyr-4 also induced Egr-1 in breast cancer cells, and RNA interference assays showed that these responses were dependent on COUP-TFI and the Sp proteins (Sp1 and Sp3). DIM-C-Pyr-4 is the first example of a compound that activates COUP-TFI and induced COUP-TFI-dependent Egr-1 gene expression in breast cancer cells, and these responses are due, in part, to activation of phosphatidylinositol-3-kinase (PI3-K) and cyclic adenosine monophosphate (cAMP)/protein kinase A (PKA).

## 2. Materials and Methods

### 2.1. Cell Lines, Chemicals, and Reagents

The MCF-7 and ZR-75 breast cancer cell lines were obtained from the American Type Culture Collection (ATCC, Manassas, VA, USA). Cells were maintained in Roswell Park Memorial Institute (RPMI) 1640 media (Sigma, St. Louis, MO, USA) supplemented with 10% fetal bovine serum (FBS) (Summit Biotechnology, Fort Collins, CO, USA; Intergen, Des Plains, IA, USA; JRH Biosciences, Lenexa, KS, USA; or Atlanta Biologicals, Inc., Norcross, GA, USA). Medium was further supplemented with 1.5 g/L sodium bicarbonate, 2.38 g/L HEPES, 0.11 g/L sodium pyruvate, and 100× antibiotic/antimycotic solution (Sigma). Cells were maintained at 37 °C with a humidified CO2:air (5:95) mixture. The GAL4 reporter containing 5× GAL4-DBD (GAL4-luc) was kindly provided by Dr. Marty Mayo (University of North Carolina, Chapel Hill, NC, USA). The GAL4-coactivator fusion plasmids pM-steroid receptor coactivator-1 (SRC-1), pM-amplified in breast cancer-1 (AIBI), pM-TIFII, pM-vitamin D-interacting protein 205 (DRIP205), pM-thyroid hormone receptor-associated protein 220 (TRAP220), and pM-coactivator-associated arginine methyltransferase-1 (CARM-1) were kindly provided by Dr. Shigeaki Kato (University of Tokyo, Tokyo, Japan). pM is the empty vector control. The dominant negative PKA plasmid was supplied by Dr. Richard Maurer (Oregon Health Sciences Center, Portland, OR, USA). Horseradish peroxidase substrate for Western blot analysis was purchased from NEN Life Science Products (Boston, MA, USA). H89, SQ22536, wortmannin, and LY294002 were purchased from Calbiochem (La Jolla, CA, USA). Both SP600125 and GF109203X were purchased from Sigma–Aldrich (St. Louis, MO, USA). Cell lysis buffer and luciferase reagent were purchased from Promega (Madison, WI, USA), and β-galactosidase reagent was from Tropix (Bedford, MA, USA). The heteroaromatic C-DIM compounds were essentially prepared as previously described [35]. Four mmole of the corresponding indole or substituted indole was incubated with 2 mmole of the heteroaromatic aldehyde in aqueous acetic acid (pH 2.0) for up to 10 d. The progress of the reaction was monitored by thin-layer chromatography. The resulting mixture was filtered, dried, and recrystallized from benzene/hexane to give the required products which were > 98% pure as determined by gas chromatography alone or coupled to a mass spectrometer. Indole, 1-methylindole, 2-methyl¬indole, 5-methylindole, 4-pyridinecarboxaldehyde N-oxide, pyridine-4-carboxaldehyde, pyridine-3-carboxaldehyde, 2-furaldehyde, pyridine-2-carboxaldehyde, indole-3-carboxaldehyde, 2-thiophene-carboxaldehyde, 5-phenyl-2-furaldehyde, 3-thiophene-carboxaldehyde, 3-thiophene- carboxaldehyde, 5-phenyl-2-thiophene-carboxaldehyde, pyrrole-2-carboxaldehyde, and 5-(3-trifluoromethyl)phenylfurfural were all purchased from Aldrich (Milwaukee, WI, USA). The heteroaromatic C-DIMs used in this study included: 1,1-bis(3′-indolyl)-1-(4-pyridyl)methane (DIM-C-Pyr-4), 1,1-bis(3′-indolyl)-1-(3-pyridyl)-methane (DIM-C-Pyr-3), 1,1-bis(3′-indolyl)- 1-(2-pyridyl)methane (DIM-C-Pyr-2), 1,1-bis[3′-(1-methylindolyl)]-1-(4-pyridyl)methane (1,1-diMe), 1,1-bis[3′-(2-methylindolyl)]-1-(4-pyridyl)-methane (2,2-diMe), 1,1-bis[3′-(5-methylindolyl)]-1- (4-pyridyl)methane (5,5-diMe), 1,1-bis(3′-indolyl)-1-(4-pyridyl N-oxide)methane (N-O), 1,1- bis(3′-indolyl)-1-(2-furanyl)methane (furan), 1,1-bis(3′-indolyl)-1-(2-thiophenyl)methane(thio- phene-2), 1,1-bis(3′indolyl)-1-[2-(5-phenyl-furanyl)]methane (5-Ph-furan-2), 1,1-bis(3′-indolyl)-1-(3- thiophenyl)methane (thiophene-3), 1,1-bis(3′-indolyl)-1-[5-(3-trifluoromethylphenyl)furfuryl] methane (furan-PhCF3), 1,1-bis(3′indolyl)-1-(2-pyrrolyl)methane (pyrrole-2), 1,1-bis(3′-indolyl)-1-[3- (5-phenylthiophenyl)]methane (5-Ph-thiophene-3), and 1,1-bis(3′-indolyl)-1-(3-indolyl)methane (indole-3).

### 2.2. Transfection Assays

MCF-7 cells were plated in 12-well plates at 1.5 × 10^5^ cells/well in Dulbecco’s Modified Eagle’s Medium/Nutrient Mixture F-12 (DME/F-12) media supplemented with 2.5% charcoal-stripped fetal bovine serum. ZR-75 cells were plated in RPMI-1640 media supplemented with 2.5% charcoal-stripped fetal bovine serum. The next day, various amounts of DNA, i.e., GAL4-Luc (0.4 μg), β-gal (0.05 μg), VP-COUP-TFI or VP-COUP-TFI-ΔN (0.05 μg), pM-SRC1 (0.05 μg), pM-SRC2 (0.05 μg), pM-SRC3 (0.05 μg), pM-TRAP220 (0.05 μg), pM-PGC-1 (0.05 μg), pM-SMRT (0.05 μg), and pM-CARM-1 (0.05 μg) were transfected by Transfast (Promega Corp., Madison, WI, USA) according to the manufacturer’s protocol. Twenty-four h after transfection, cells were treated with vehicle control (Me_2_SO) or the indicated amount of compound for 18–24 h. For the inhibitor study, cells were pretreated with inhibitors for 30 min and further incubated with the indicated amount of compound for 12 h. Cells were then lysed with 1× reporter lysis buffer (Promega Corp.), and luciferase activity was determined and normalized to β-galactosidase activity using a luciferase assay system (Promega Corp.) 

### 2.3. Chromatin Immunoprecipitation (ChIP) Assay

MCF-7 cells (1 × 10^7^) were treated with Me_2_SO (time 0), C-DIM-Pyr-4 (5 μM) for 15, 30, and 60 mins. Cells were then fixed with 1.5% formaldehyde, and the cross-linking reaction was stopped by addition of 0.125 M glycine. Cells were scraped, pelleted, and hypotonically lysed, and nuclei were collected. Nuclei were then sonicated to desired chromatin length (~500 bp). The chromatin was precleared by addition of protein A/G-conjugated beads (Pierce, Rockford, IL, USA). The pre-cleared chromatin supernatants were immunoprecipitated with antibodies specific to IgG, TFIIB, SRC-1, SRC-3, Sp1, Sp3, Sp4, and COUP-TFI (Santa Cruz Biotechnology, Santa Cruz, CA, USA) at 4 °C overnight. The protein-antibody complexes were collected by addition of protein A/G-conjugated beads (Pierce, Rockford, IL, USA) for 1 h, and the beads were extensively washed. The protein-DNA crosslinks were eluted and reversed. The DNA was purified by Qiaquick Spin Columns (Qiagen, Valencia, CA, USA) followed by PCR amplification. The Egr-1 primers were: 5′-CCT AGG GTG CAG GAT GGA G-3′ (forward), and 5′-GGA TCC GCC TCT ATT TGA AG-3′ (reverse), which amplified a 172-bp region of the human Egr-1 promoter containing a proximal GC-rich region (−64~−46). The positive control primers were: 5′-TAC TAG CGG TTT TAC GGG CG-3′ (forward), and 5′-TCG AAC AGG AGG AGC AGA GAG CGA-3′ (reverse), which amplified a 167-bp region of human glyceraldehyde-3-phosphate dehydrogenase (GAPDH) gene. The negative control primers were: 5′-ATG GTT GCC ACT GGG GAT CT-3′ (forward), and 5′-TGC CAA AGC CTA GGG GAA GA-3′ (reverse), which amplified a 174-bp region of genomic DNA between the GAPDH and CNAP1 genes. The PCR products were resolved on a 2% agarose gel in the presence of 1:10 000 SYBR gold (Molecular Probes, Eugene, OR, USA).

### 2.4. RNA Interference Studies

MCF-7 and ZR-75 breast cancer cells were cultured in phenol red-free DME/F12 medium supplemented with 2.5% charcoal stripped FBS in 6-well plates and were 50% confluent for the experiments. All small interfering RNA (siRNA) oligonucleotides were diluted in Opti-MEM (InVitrogen, Carlsbad, CA, USA) for a final concentration of 20 nM and transfected using Lipofectamine 2000 reagent (InVitrogen) according to the manufacturer’s protocol. After incubation for 60 h, cells were harvested and the efficiency of protein knockdown by siRNAs was determined by Western blot using whole cell (ZR-75) or nuclear (MCF-7) extracts which were obtained using the NE-PER nuclear and cytosolic extraction kit (Pierce) according to the manufacturer’s instructions. The siRNA oligonucleotides for Sp1, Sp3, and Sp4 were obtained from Dharmacon (Lafayette, CO, USA) as follows: Sp1, 5′-AUC ACU CCA UGG AUG AAA UGA dTdT-3′; Sp3, 5′-GCG GCA GGU GGA GCC UUC ACU dTdT-3′; and Sp4, 5′-GCA GUG ACA CAU UAG UGA GCdT dT-3′; siRNA oligonucleotide for COUP-TFI obtained from InVitrogen as follow: 5′-AAA CGG ACG AAG AAG AGC UGC UCG A-3′, and non-target negative control siRNA oligonucleotide was purchased from Qiagen (Valencia, CA, USA).

### 2.5. Induction of Egr-1 Protein/mRNA by DIM-C-Pyr-4

Induction of Egr-1 protein by DIM-C-Pyr-4 was determined by Western blot assay. MCF-7 and ZR-75 cells were seeded in 6-well plates in DME/F-12 medium without phenol red containing 2.5% charcoal-stripped FBS. After 24 h, cells were treated with 5.0 μM DIM-C-Pyr-4, harvested at designated time points and lysed in ice-cold lysis buffer (50 mM HEPES (pH 7.5), 500 mM NaCl, 10% (*v*/*v*) glycerol, 1% Triton X-100, 1.5 mM MgCl2, 1 mM ethylene glycol tetraacetic acid (EGTA) supplemented with protease inhibitor cocktail (Sigma). Equal amounts of protein from each treatment group were resolved by 10% polyacrylamide gel electrophoresis (SDS-PAGE), and transferred to a polyvinylidene difluoride (PVDF) membrane. Membranes were blocked with 5% skim milk and then probed with Egr-1 antibodies (Cell Signaling Technology, Beverly, MA, USA). Following incubation with peroxidase-conjugated secondary antibody, immunoglobulins were visualized using the ECL detection system (Perkin Elmer, Foster City, CA, USA). The follow antibodies were used in the RNA interference experiments: Sp1, Sp3, and Sp4 (Santa Cruz Biotechnology Inc., Santa Cruz, CA, USA); COUP-TFI (R&D Systems, Inc., Minneapolis, MN, USA); α/β-tubulin and histone H3 (Cell Signaling Technology, Beverly, MA, USA).

Induction of Egr-1 mRNA by DIM-C-Pyr-4 was determined by real-time PCR assay. Total RNA was isolated from MCF-7 and ZR-75 cells using RNeasy Protect Mini Kit (Qiagen) according to the manufacturer’s protocol. RNA was reverse transcribed using Superscript III reverse transcriptase (Invitrogen) according to the manufacturer’s protocol. The PCR was carried out using SYBR Green PCR Master Mix (PE Applied Biosystems, Warrington, UK) on an ABI Prism 7700 Sequence Detection System (PE Applied Biosystems). TATA-binding protein (TBP) was used as an endogenous control to compare the relative amount of target gene in different samples. The PCR experiment was carried out as follows: 1 cycle of 95 °C for 10 min, then 40 cycles of 95 °C for 15 s, and 60 °C for 1 min. The comparative cycle threshold (Ct) method was used for relative quantitation of samples. The primer specific amplifying a 97 bp of human Egr-1 transcript was purchased from Qiagen.

### 2.6. Clones and Reagents

The wild-type mouse COUP-TFI expression vector (pCR3.1-COUP-TFI) was originally provided by Dr. Ming-Jer Tsai (Baylor College of Medicine, Houston, TX, USA) and used as the PCR template for further cloning. The COUP-TFI expression plasmids used in the transfection assay including WT, N-terminal deletion mutants (dN1, and dN2), and C-terminal deletion mutants (dC1 and dC2) were generated by PCR amplification and ligated into pCDNA3.1/His or pCDNA3.1/HIS/-Myc vectors (Invitrogen). The GAL4-DBD-COUP-TFI and VP-16-COUP-TFI chimeras expression plasmid were also generated by PCR amplification and cloned into pBIND or pACT vectors (Promega). The following primers were used for cloning the COUP-TFI constructs: COUP-TFI WT, 5′-GCG GCC GCA TGG CAA TGG TAG TTA G-3′ and 5′-GCT GCT CGA GGG AAC ACT GGA TGG ACA TGT-3′; COUP-TFI dN1, 5′-TAT AGG TAC CTG GTT CAG GCC AGA GCC AGC AGC A-3′ and 5′-GCT GCT CGA GGG AAC ACT GGA TGG ACA TGT-3′; COUP-TFI dN2, 5′-TAG CGG TAC CTA GGA GCG TCC GCA GGA ACT TAA C-3′ and 5′-GCT GCT CGA GGG AAC ACT GGA TGG ACA TGT-3′; COUP-TFI dN3, 5′-TCT AGA ATG GAA GCG GTT CAG CGA GGA A-3′ and 5′-GCG GCC GCC TAG GAA CAC TGG ATG G-3′; COUP-TFI dC1 5′-AGC TAA GCT TAT GGC AAT GGT AGT TAG CAG-3′ and R, 5′-AGC TCT CGA GCA GCA GTT TGC CAA AGC GGC-3′; COUP-TFI dC2 5′-AGC TAA GCT TAT GGC AAT GGT AGT TAG CAG-3′ and 5′-AGC TCT CGA GCA GCG GCA TGG AGC AC-3′.

### 2.7. Computation Analysis of COUP-TFI and COUP-TFII Ligand Binding

We computationally investigated the binding of compounds C-DIM-Pyr4 and C-1,1-CH3-DIM-Pyr4 to human proteins COUP-TFI (residues 177 through 414) and COUP-TFII (residues 171 through 407). Our investigation initially focused on elucidating the binding of the two compounds to COUP-TFII, for which previous experimental site-directed mutagenesis studies mapping its key residues associated with activity were available [39]. As a result, we considered that this information could serve as a means to validate our computational studies. Hence, the validated computational results in conjunction with previous experimental data could be used to provide in-depth insights into the structural and energetic determinants associated with activity of COUP-TFII. In addition, given the very high sequence similarity between COUP-TFI and COUP-TFII (the two proteins are nearly identical sharing 97% identity within the domains mentioned above), we considered that our computationally validated studies on COUP-TFII could be used as an excellent starting point to model the interactions between the same compounds in complex with COUP-TFI.

To model the structure of COUP-TFII in complex with the two compounds under investigation, COUP-TFII residues (171 through 407) were extracted from the crystal structure of the human COUP-TFII ligand-binding domain (PDB ID: 3CJW) [39] with loop residues 194–206 and 269–285 introduced through MODELLER [40]. The structure of COUP-TFII shows that the binding site is densely packed with aromatic and hydrophobic residues (I212, A216, L220, W249, F253, A257, M262, F295, I378, F382, F383, I392), leaving a cavity less than 20 Å^3^ in volume [39]. To refine the initial structure and to allow the COUP-TFII binding pocket to potentially expand, and thus facilitate the initial, independent placement of the two compounds, we introduced a short 10 ns constrained explicit-solvent molecular dynamics (MD) simulation in which binding pocket residues (207–223, 245–300, 370–396) and the modeled loop residues (194–206, 269–285) were unconstrained and the remaining residues were lightly constrained with 1.0 kcal/mol on heavy backbone atoms and 0.5 kcal/mol on heavy side chain atoms. The MD simulation was performed in a 109 Å cubic water box with the N- and C-terminal ends of the modeled protein acetylated and amidated to eliminate artificially placed positive and negative charges at the backbone termini analogously to References [41,42,43,44,45,46,47].

Using the 10 ns conformation of the un-complexed COUP-TFII within the simulation, we initially independently placed C-DIM-Pyr4 and C-1,1-CH3-DIM-Pyr4 into the binding pocket of COUP-TFII using AutoDock Vina [48] and employed our in-house developed docking protocol [43,44,45,49] to perform a highly-detailed search of the possible binding modes of the two compounds within the binding pocket, and thus uncover the binding modes of the two compounds in complex with COUP-TFII with the highest energetic favorability. In summary, the protocol consists of: (1) the use of multiple short MD docking simulations nearly exhaustively search binding conformations of the compound within the receptor binding pocket with the compound constrained to the binding pocket though harmonic and quartic spherical potentials and the binding pocket of the receptor unconstrained; (2) the selection of sets of the most probable binding modes generated by the short docking simulations based on interaction energy calculations; and (3) the use of explicit-solvent MD simulations and physical-chemistry based free energy calculations to identify the most favorable binding mode of the compound:receptor complex.

Here, six separate docking simulation systems were introduced independently to explore the possible binding modes of the two compounds. During the docking simulations, the compound was constrained to the binding pocket using quartic and harmonic spherical potentials through the miscellaneous mean field potential (MMFP) module of chemistry at Harvard macromolecular mechanics (CHARMM) [50] and the binding pocket residues (207–223, 245–300, 370–396) were unconstrained to facilitate the compounds’ exploration of the binding pocket. Four of the six docking simulations systems employed a harmonic spherical potential. Two of the six docking simulation systems employed a quartic potential energy function, which energetically encourages the compound to explore binding modes away from its initial positioning, and thus reduces the bias introduced by the initial placement of the compounds. In each of the six docking simulation systems, 20 independent runs comprising 200 steps of short 2 ps simulations were performed. In each of the 200 steps, prior to the 2 ps MD simulation, the compound was rotated about a randomly generated axis, and subsequent to the short MD simulation, minimized and saved for evaluation. This procedure resulted in the generation of 4000 binding modes of each compound in complex with COUP-TFII per docking simulation system. Of the 4000 binding modes produced per docking simulation system, the three binding modes with the lowest interaction energy were selected for further investigation, resulting in a total of 18 extracted binding modes of C-DIM-Pyr4 and C-1,1-CH3-DIM-Pyr4 in complex with COUP-TFII. The 18 binding modes of C-DIM-Pyr4 and C-1,1-CH3-DIM-Pyr4 were investigated through 20 ns explicit-solvent MD simulations to refine the compound:receptor structures, optimize intermolecular interactions, determine the structural stability of the selected binding modes, and determine the most energetically favorable binding modes. All MD simulations were performed in explicit-solvent using CHARMM [50] and CHARMM36 topologies and parameters [51] with periodic boundary conditions. The energetic favorability of the binding modes was assessed using molecular mechanics generalized born surface area (MM-GBSA) association free energy calculations as previously described [43,44,45,48]. Additional details on the docking-refinement protocol are provided in Reference [43]. 

From the 18 binding modes of the C-DIM-Pyr4:COUP-TFII complex and the 18 binding modes of the C-1,1-CH3-DIM-Pyr4:COUP-TFII complex, we independently identified the complex structures with the lowest, most favorable, average MM-GBSA association free energies. The simulation encompassing the most energetically favored binding mode of C-DIM-Pyr4:COUP-TFII was derived from a docking simulation at which the compound was maintained in the binding pocket using a quartic potential energy function while the simulation encompassing the most energetically favored binding modes of C-1,1-CH3-DIM-Pyr4:COUP-TFII was derived from a docking simulation at which the compound was maintained in the binding pocket using a harmonic potential energy function. For each of the two complexes under investigation, the simulated binding modes identified were, independently, at least 5 kcal/mol more energetically favorable than all other simulated binding modes of the same complex, according to the MM-GBSA association free energy calculations; thus, they likely correspond to the naturally occurring binding conformations. The simulation corresponding to the most energetically favorable binding mode for C-DIM-Pyr4 in complex with COUP-TFII, and C-1,1-CH3-DIM-Pyr4 in complex with COUP-TFII extended to additional 10 ns, thus reaching 30 ns each and studied as described below.

The root mean square deviation (RMSD) of C-DIM-Pyr4 and C-1,1-CH3-DIM-Pyr4 heavy atoms with respect to their initial structure is 4.3 ± 1.0 Å and 3.3 ± 0.4 Å, respectively, indicating the importance of MD simulations in refining the initial conformation of the compound:receptor complex structures, especially within the first 10 ns of the simulations. The stability of the compounds in the COUP-TFII binding pocket was determined through RMSD calculations of the compound heavy atoms with respect to their average conformation within their simulation trajectories. The RMSD of C-DIM-Pyr4 and C-1,1-CH3-DIM-Pyr4 heavy atoms with respect to the average structure over the last 20 ns of the MD simulations is 1.3 ± 0.5 Å and 0.9 ± 0.5 Å, respectively, indicating that the binding of the two compounds is stable within the binding pocket. As both compounds readjust their positions within the first 5 ns of their respective simulations, we treated the first 10 ns, in addition to the 1 ns constrained equilibration stage prior, of the total 30 ns MD simulation as additional equilibration and focused our analysis on the final 20 ns of the MD simulation. The key interactions occurring within the selected complex structures were determined through the calculation of average per COUP-TFII residues interaction free energies to each compound using the final 20 ns of the MD simulations. Additional information on the calculations calculating interaction free energy per COUP-TFII residue are provided in Reference [43].

**Figure 1:**
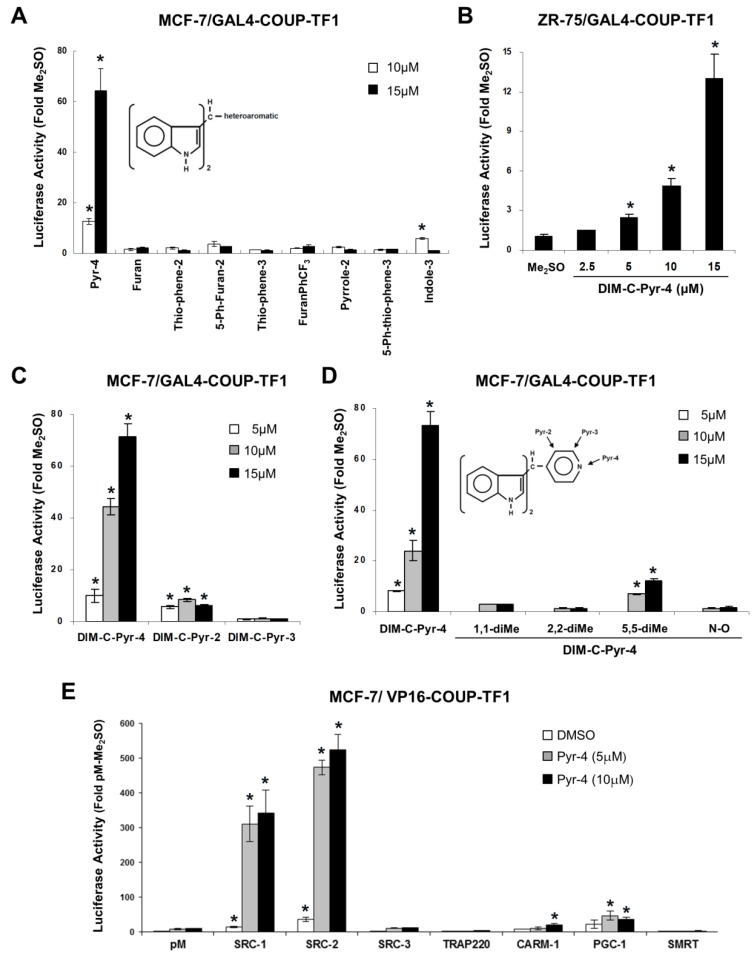
Heteroaromatic methylene substituted diindolylmethanes (C-DIMs) activate Chicken ovalbumin upstream promoter-transcription factor I (COUP-TFI) and induce COUP-TFI-coactivator interactions. Activation of GAL4-COUP-TFI in MCF-7 (**A**,**C**,**D**) and ZR-75 (**B**) cells by heteroaromatic C-DIMs. Cells were transfected with GAL4-COUP-TFI and GAL4-luc constructs treated with Me_2_SO (control) or different concentrations of heteroaromatic C-DIMs, and luciferase activity (relative to β-galactosidase activity) was determined as described in the Materials and Methods section. Results are means ± SE for three replicate determinations for each treatment group and significant (*p* < 0.05) induction is indicated by an asterisk. (**E**) Mammalian two-hybrid assay. MCF-7 cells were transfected with VP-COUP-TFI/GAL4-luc and chimeric GAL4-coactivator constructs, treated with Me_2_SO, 10 or 15 μM 1,1-bis(3′-indolyl)-1-(4-pyridyl)-methane (DIM-C-Pyr-4), and luciferase activity determined as described in the Material and Methods section. Results are expressed as means ± SE for three replicate determinations for each treatment group and significant (*p* < 0.05) induction is indicated by an asterisk.

Given the high sequence similarity of COUP-TFI and COUP-TFII, both within the overall residue domains included in the simulation system (97%), and especially within the binding pockets of the two compounds which are identical (Figure 1 and Figure S1), we used the insights from the predicted simulation structures of the two compounds in complex with COUP-TFII, to computationally investigate the corresponding complexes with COUP-TI. Using the binding modes of C-DIM-Pyr4 and C-1,1-CH3-DIM-Pyr4 in complex with COUP-TFII that result in the most energetically favorable complex structures as a basis, we obtained the corresponding conformations for the COUP-TFI complexes. Appropriate mutations from COUP-TFII to COUP-TFI residues were introduced using SCWRL4.0 [52]. Subsequently, we performed 30 ns MD simulations of the modeled COUP-TFI in complex with the two compounds independently. Similar to the simulations of the COUP-TFII complexes, the structures of both C-DIM-Pyr4 and C-1,1-CH3-DIM-Pyr4 in the complex with COUP-TFI were refined within the 30 ns simulations (3.6 ± 0.9 and 2.8 ± 0.4 RMSD of their heavy atoms with respect to their initial structure, respectively). Both compounds were also stable within the COUP-TFI binding pocket (1.1 ± 0.4 and 0.8 ± 0.3 RMSD of their heavy atoms with respect to their average structure over the last 20 ns of the simulations, respectively). Upon completion of the simulations, we extracted the simulation trajectory snapshots corresponding to the last 20 ns and analyzed the key interactions occurring within the complex structures of COUP-TFI in complex with the two compounds, using similar approaches to what is described above for COUP-TFII.

### 2.8. Statistical Analysis

Statistical differences between different groups were determined by *t*-test for significance. The data are presented as mean ± SE for at least three separate determinations for each treatment.

## 3. Results

### 3.1. Structure-Dependent Activation of COUP-TFI and Interaction with Coactivators and Silencing Mediator of Retinoic Acid and Thyroid Hormone Receptor (SMRT)

Studies in this laboratory on a series of C-DIM compounds containing various substituted phenyl groups have shown that some of these analogs have been identified as agonists for the orphan receptors PPAR and nuclear receptor subfamily 4, group A, member 1 (NR4A1, Nur77) [35,36,37,38]. A series of C-DIMs containing heteroaromatic substituents was also prepared, and the results in Figure 1A summarize the effects of heteroaromatic C-DIMs on activation of COUP-TFI in MCF-7 breast cancer cells transfected with GAL4-COUP-TFI (full length) and a reporter construct (GAL4-luc) containing 5 tandem GAL4 response elements. This series of C-DIMs contained 3-indole, 3-(5-phenylthiophene), 2-pyrrole, 5-(3′-trifluoromethylphenylfuran, 3-thiophene, 2-(5-phenylfuran), 2-thiophene, 2-furan, and 4-pyridine heteroaromatic substituents, and only the latter compound induced a >60-fold increase in luciferase activity. The substituted phenyl C-DIM analogs (35–38) were also inactive in this assay (data not shown). The 3-indole substituted analog induced minimal activity and the other compounds were inactive. The DIM-C-Pyr-4 derivative also activated GAL4-COUP-TFI in ZR-75 cells (Figure 1B), and dose–response studies in ZR-75 and MCF-7 cells showed that induction was observed at 2.5–5.0 μM concentrations. The structure-dependent activation of GAL4-COUP-TFI was determined with the 3-pyridyl (DIM-C-Pyr-3) and 2-pyridyl (DIM-C-Pyr-2) isomers (Figure 1C) and a series of DIM-C-Pyr-4 analogs containing 1,1-dimethyl, 2,2’-dimethyl, 5,5′-dimethyl, and N-oxide substituents (Figure 1D). DIM-C-Pyr-2 and the 5,5′-dimethyl analog of DIM-C-Pyr-4 exhibited some activity and the other compounds were inactive, suggesting that DIM-C-Pyr-4 was the most potent of the pyridyl substituted analogs for activation of GAL4-COUP-TFI. 

The intracellular location of COUP-TFI was determined by immunocytochemistry and nuclear staining was observed in control (Me_2_SO) and DIM-C-Pyr-4 treated cells (data not shown). Ligand-dependent interactions of COUP-TFI with coactivators was also determined in MCF-7 cells transfected with GAL4-coactivator/VP-COUP-TFI chimeras and pGAL4-luc. Results of this mammalian two-hybrid assay (Figure 1E) showed that DIM-C-Pyr-4 primarily induced COUP-TFI interactions with SRC-1 and SRC-2, whereas minimal (CARM-1 or PGC-1) or no (SRC-3, TRAP220) interactions were observed with other coactivators or the corepressor SMRT. Thus, DIM-C-Pyr-4-dependent interactions of COUP-TFI with coactivators in MCF-7 cells was highly selective; moreover, a previous study showed that in the absence of exogenous compounds, SRC-1 and SRC-2 interacted with COUP-TFI and enhanced COUP-TFI-mediated transactivation in HeLa cells [29], and this was also observed in MCF-7 cells (Figure 1E).

### 3.2. Activation of Variant GAL4-COUP-TFI Chimeras by DIM-C-Pyr-4 and Related Compounds

**Figure 2:**
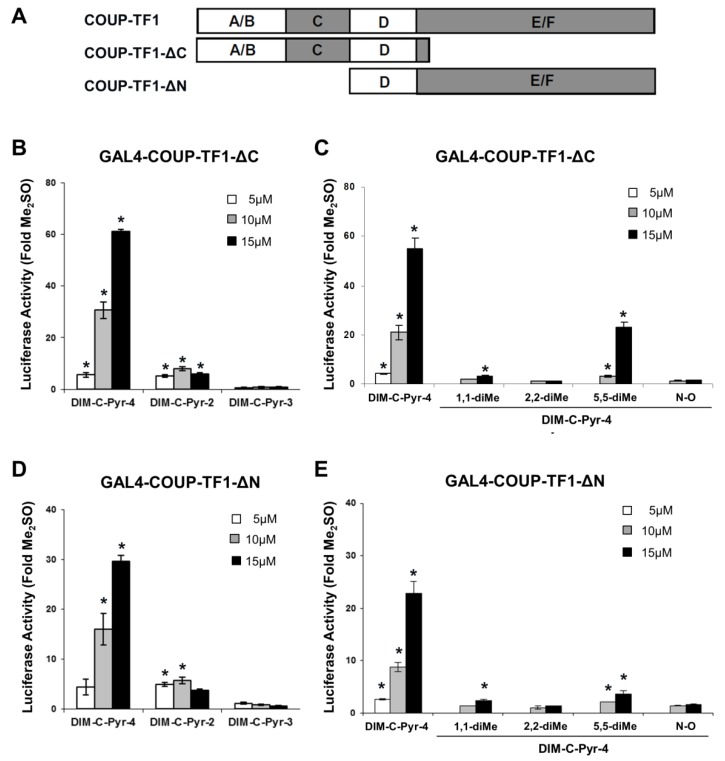
Activation of COUP-TFI domains by heteroaromatic C-DIMs. (**A**) Wild-type COUP-TFI and deletion variants. MCF-7 cells were transfected with GAL4-COUP-TFI-ΔC (**B**,**C**) or GAL4-COUP-TFI-ΔN (**D**,**E**) and GAL4-luc, treated with Me_2_SO or heteroaromatic C-DIMs, and luciferase activity determined as described in the Materials and Methods section. Results are expressed as means ± SE for three replicate determinations for each treatment group and significant (*p* < 0.05) induction is indicated by an asterisk.

Nuclear receptors such as COUP-TFI exhibit common structural features (Figure 2A) which include C-terminal and N-terminal activation function 1 (AF1) and AF2, respectively, and the ligand binding domain in the C-terminal (E/F) region of the receptor. DIM-C-Pyr-4 activated GAL4-COUP-TFI-ΔC (Figure 2B,C) and COUP-TFI-ΔN (Figure 2C,D) which express the N- and C-terminal regions of COUP-TFI, respectively. Further examination of the hinge (D) region was not determined since previous studies on GAL4-COUP-TF1 deletion mutants showed that this region had minimal effects on transactivation [29]. The structure activity relationships for activation of these constructs by the different DIM-C-pyridyl analogs were similar and also corresponded to the structure-dependent activation of wild-type GAL-COUP-TFI (Figure 1C,D). Activation of nuclear receptors is complex and is typically due to both interactions with agonists and activation of kinase pathways which are particularly important for AF1-mediated responses [53,54,55,56,57]. 

**Figure 3:**
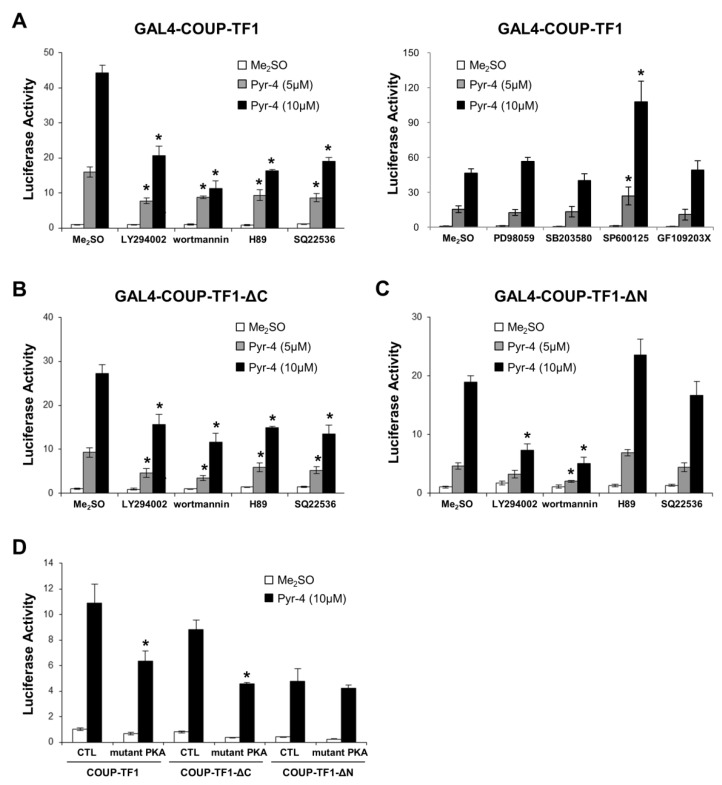
Role of kinases in activation of COUP-TFI by DIM-C-Pyr-4. MCF-7 cells were transfected with GAL4-luc and GAL4-COUP-TFI (**A**), GAL4-COUP-TFI-ΔC (**B**), GAL4-COUP-TFI-ΔN (**C**), or all three constructs (**D**), treated with Me_2_SO or DIM-C-Pyr-4 alone or in the presence of 10 μM LY294002, 500 nM wortmannin, 10 μM H89, 400 μM SQ22536, 20 μM PD98059, 20 μM SB203580, 20 μM SP600125, 5 μM GF109203X or transfected dominant negative PKA expression plasmid, and luciferase activity was determined as described. Results are expressed as means ± SE for three replicate determinations for each treatment group, and significantly (*p* < 0.05) decreased activity in cells cotreated with kinase inhibitors or dominant negative PKA (compared to cells treated with DIM-C-Pyr-4 alone) is indicated by an asterisk.

**Figure 4:**
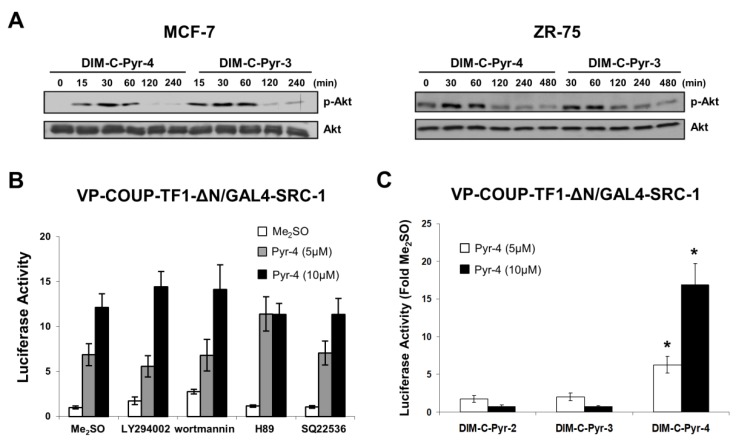
Ligand-induced interactions of the C-terminal domain of COUP-TFI with SRC-1. (**A**) Activation of PI3-K by DIM-C-Pyr-4 and DIM-C-Pyr-3. MCF-7 or ZR-75 cells were treated with 15 μM DIM-C-Pyr-4 or DIM-C-Pyr-3 for up to 480 min, and phospho-Akt and Akt protein expression was determined by Western blot analysis of whole-cell lysates as described in the Materials and Methods section. (**B**) Effects of kinase inhibitors on DIM-C-Pyr-4-induced SRC-1-COUP-TFI-ΔN interactions. MCF-7 cells were transfected with GAL4-SRC-1/GAL4-luc and VP-COUP-TFI-ΔN, treated with Me_2_SO or DIM-C-Pyr-4 alone or in combination with kinase inhibitors as described above, and luciferase activity was determined as described in the Materials and Methods section. Results are expressed at means ± SE for three replicate determinations for each treatment group. (**C**) Structure-dependent interactions of GAL4-SRC-1 and COUP-TFI-ΔN. MCF-7 cells were transfected as described in (**B**), treated with Me_2_SO, DIM-C-Pyr-4, DIM-C-Pyr-3 or DIM-C-Pyr-2, and luciferase activity determined as described in the Materials and Methods section. Results are means ± SE for triplicate determinations, and significant (*p* < 0.05) induction is indicated by an asterisk.

Based on the assumption that DIM-C-Pyr-4 may act as a COUP-TFI agonist and also activate kinase pathways, we investigated the effects of several kinase inhibitors on luciferase activity in MCF-7 cells transfected with pGAL4-luc and GAL4-COUP-TFI, GAL4-COUP-TFI-ΔC or GAL4-COUP-TFI-ΔN (Figure 3A–C). MEK inhibitor (PD98059), p38 MAP kinase inhibitor (SB203580), and PKC inhibitor (GF109203X) did not inhibit transactivation in cells transfected with GAL4-COUP-TFI (Figure 3A). JNK inhibitor, SP600125 enhanced basal and ligand-induced transactivation; however, the fold induction was not observed with GAL4-COUP-TFI. The results showed that only the PI3-K inhibitors wortmannin and LY294002 and cAMP/PKA inhibitors H89 and SQ22536 inhibit transactivation with GAL4-COUP-TFI and GAL4-COUP-TFI-ΔC (Figure 3A,B). These results suggest that DIM-C-Pyr-4 activates both the PI3-K and cAMP/PKA pathways to enhance AF1, and this significantly contributes to activation of COUP-TFI. In contrast, PI3-K but not cAMP/PKA inhibitors block activation of GAL4-COUP-TFI-ΔN (Figure 3C), and the specificity of the PKA pathway for activation of the N-terminal region of COUP-TFI was confirmed using a dominant negative PKA expression plasmid which inhibited activation of GAL4-COUP-TFI, GAL4-COUP-TFI-ΔC but not GAL4-COUP-TFI-ΔN (Figure 3D). The chimera containing the ligand binding domain (GAL4-COUP-TFI-ΔN) was significantly activated by DIM-C-Pyr-4, even in cells cotreated with PI3-K inhibitors suggesting that this response may be due, in part, to COUP-TFI agonist activity, activation by an identified kinase or both. Therefore, we further investigated the role of DIM-C-Pyr-4 in activation of COUP-TFI by first comparing the activation of PI3-K by this compound and an inactive analog DIM-C-Pyr-3. The results show that both DIM-C-Pyr-4 and DIM-C-Pyr-3 induce PI3-K-dependent phosphorylation of Akt (Figure 4A). Since DIM-C-Pyr-4 but not DIM-C-Pyr-3 activates GAL4-COUP-TFI (Figure 1), the results in Figure 4A indicate that induction of PI3-K-dependent phosphorylation of Akt was not sufficient for activation of GAL4-COUP-TFI. The potential role of DIM-C-Pyr-4 as a COUP-TFI agonist was further investigated in a mammalian two-hybrid assay in MCF-7 cells transfected with VP-COUP-TFI-ΔN and GAL4-SRC-1 in the absence (Me_2_SO) or presence of PI3-K (LY294002 and wortmannin) and cAMP/PKA (H89 and SQ22536) inhibitors (Figure 4B). Although, the PI3-K inhibitors increase transactivation in cells treated with Me_2_SO, only minimal effects were observed on luciferase activity induced by DIM-C-Pyr-4. Moreover, a direct comparison of the effects of DIM-C-Pyr-4 with the inactive DIM-C-Pyr-3 and DIM-C-Pyr-2 analogs in the mammalian two-hybrid assay shows that only the former compound induces SRC-1-COUP-TFI-ΔN interactions in the mammalian two-hybrid assay (Figure 4C). These results indicate that DIM-C-Pyr-4-induced interactions of the ligand binding domain of COUP-TFI with SRC-1 was not totally dependent on PI3-K and the differences observed in the effects of DIM-C-Pyr3 and DIM-C-Pyr-4 were structure-dependent.

### 3.3. DIM-C-Pyr-4 Activation of Egr-1 Is Dependent on COUP-TFI and Sp Proteins

**Figure 5:**
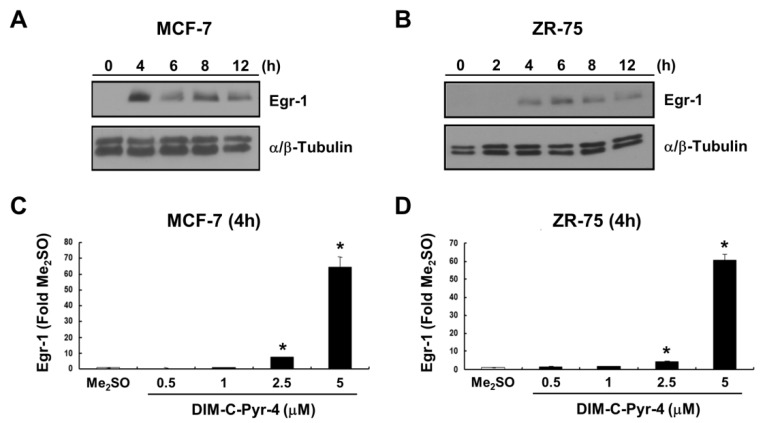
Induction of Egr-1 by DIM-C-Pyr-4. (**A**,**B**) Western blot analysis. MCF-7 (**A**) and ZR-75 (**B**) cells were treated with 5 μM DIM-C-Pyr-4 for up to 12 h, and expression of Egr-1 protein was determined by Western blot analysis of whole-cell lysates as described in the Materials and Methods section. α/β-Tubulin served as loading control. Induction of Egr-1 mRNA by DIM-C-Pyr-4 in MCF-7 (**C**) and ZR-75 (**D**) cells. Cells were treated with different concentrations of DIM-C-Pyr-4 for 4 h, and Egr-1 mRNA levels were determined as described in the Materials and Methods section. Results are expressed as means ± SE for three determinations per treatment group, and significant (*p* < 0.05) induction is indicated by an asterisk.

Overexpression of COUP-TFI in rat urogenital mesenchymal (rUGM) cell lines resulted in induction of Egr-1 (27), and results in Figure 5A,B show that 5 μM DIM-C-Pyr-4 induced Egr-1 protein in MCF-7 and ZR-75 breast cancer cells, respectively. Induction was observed within 4 h and persisted for up to 12 h after treatment. DIM-C-Pyr-4 also induced a concentration-dependent increase in Egr-1 mRNA levels in MCF-7 (Figure 5C) and ZR-75 (Figure 5D) cells after treatment for 4 h. 

**Figure 6:**
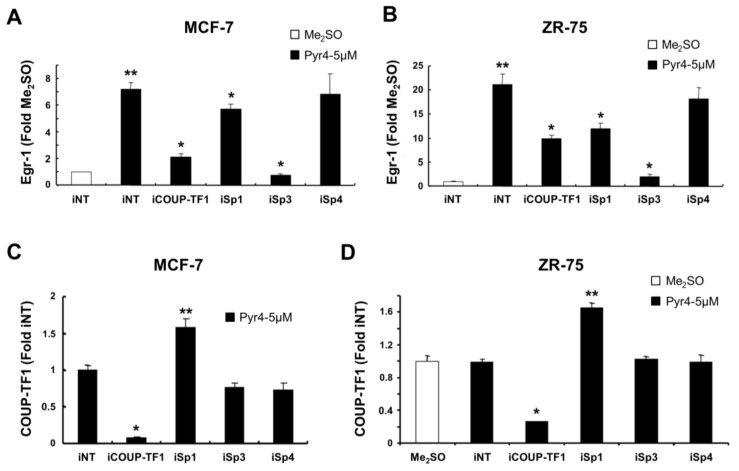
RNA interference studies. Expression of Egr-1 mRNA in MCF-7 (**A**) and ZR-75 (**B**) cells and expression of COUP-TFI mRNA in MCF-7 (**C**) and ZR-75 cells (**D**). Cells were transfected with small inhibitory RNAs, treated with 5 μM DIM-C-Pyr-4 (or Me_2_SO) for 4 h, and the fold induction of Egr-1 (A/B) and COUP-TFI (C/D) mRNA (relative to Me_2_SO) was determined as described in the Materials and Methods section. Results are means ± SE for three replicate determinations for each treatment group. Significant (*p* < 0.05) induction (**) of Egr-1 mRNA (A/B) by DIM-C-Pyr-4 and significant inhibition (*) of Egr-1 mRNA (A/B) by iCOUP-TFI or an iSp are indicated. Significant inhibition (*) or induction (**) of COUP-TFI mRNA (C/D) by a small inhibitory RNA is also indicated. iNT is a non-specific siRNA used as a control for transfection of the targeted siRNAs.

Previous studies in rUGM cells showed that activation of Egr-1 by COUP-TFI was dependent on a proximal GC-rich site (−64 to −46) in the Egr-1 promoter and involved a COUP-TFI/Sp1 complex (27). We therefore investigated induction of Egr-1 by DIM-C-Pyr-4 in MCF-7 (Figure 6A) and ZR-75 (Figure 6C) cells using small inhibitory RNAs for COUP-TFI (iCOUP-TFI), Sp1 (iSp1), Sp3 (iSp3), and Sp4 (iSp4). The latter three siRNAs were used since all three Sp proteins have been identified in MCF-7 and ZR-75 breast cancer cells [58,59]. The results show that iSp3 was the most potent inhibitor of Egr-1 mRNA induction by DIM-C-Pyr-4, and significant inhibition was also observed after transfection with iCOUP-TFI and iSp1 but not iSp4. The results suggest that DIM-C-Pyr-4 induces Egr-1 mRNA primarily through activation of COUP-TFI/Sp3 with some effects of COUP-TFI/Sp1 and minimal contributions from Sp4. Previous studies have demonstrated the specificity of the siRNAs for Sp proteins [59], and therefore results in Figure 6C,D illustrate the effects of iCOUP-TFI, iSp1, iSp3, and iSp4 on COUP-TFI mRNA levels in MCF-7 and ZR-75 cells, respectively. iCOUP-TFI significantly decreases COUP-TFI mRNA levels, whereas iSp3 and iSp4 do not affect levels of COUP-TFI transcripts in MCF-7 (Figure 6C) or ZR-75 (Figure 6D) cells. In contrast, knockdown of Sp1 increases COUP-TFI mRNA levels in both cell lines and this is consistent with the lower activity of iSp1 in decreasing induction of Egr-1 mRNA by DIM-C-Pyr-4 (Figure 5A,B) due to increased expression of COUP-TFI. These results suggest that Sp1 repressed COUP-TFI and this must be taken into account in the interpretation of the RNA interference studies using iSp1.

**Figure 7:**
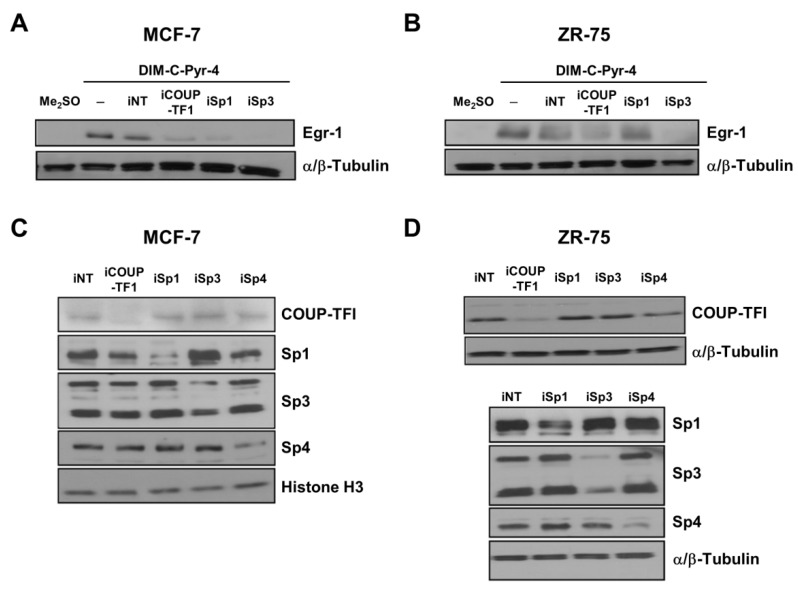
Effects of RNA interference on induction of Egr-1 protein by DIM-C-Pyr-4 and the specificity of protein knockdown. Effects of siRNAs on induction of Egr-1 protein by DIM-C-Pyr-4 in MCF-7 (**A**) and ZR-75 (**B**) cells. Cells were transfected with various siRNAs, treated with Me_2_SO or 5μM DIM-C-Pyr-4, and Egr-1 protein expression was determined by Western blot analysis of whole-cell lysates. Specificity of COUP-TFI, Sp1, Sp3, and Sp4 knockdown in MCF-7 (**C**) and ZR-75 (**D**) cells transfected with their corresponding siRNAs. Cells were transfected with iNT or various siRNAs, and COUP-TFI, Sp1, Sp3, and Sp4 protein expression was determined as described in the Materials and Methods section. α/β-Tubulin or histone H3 served as a protein loading control.

The RNA interference was also used to confirm the role of Sp proteins and COUP-TFI on the induction of Egr-1 by DIM-C-Pyr-4. Results in Figure 7A,B show that induction of Egr-1 protein decreased in MCF-7 and ZR-75 cells transfected with iCOUP-TFI and iSp3. The effects of iSp1 on induction of Egr-1 by DIM-C-Pyr-4 were dependent on cell context; in MCF-7 cells, transfection of iSp1 decreased Egr-1, whereas in ZR-75 cells, iSp1 did not affect induction of Egr-1 by DIM-C-Pyr-4. Figure 7C,D examine expression of COUP-TFI and Sp proteins in ZR-75 cells transfected with the corresponding siRNAs; the Sp proteins decreased specifically by the individual siRNAs as previously reported [59] and iCOUP-TFI decreased expression of COUP-TFI but did not affect Sp proteins. We also examined interactions of COUP-TFI, Sp proteins, SRC-1, and SRC-3 with the proximal region of the Egr-1 promoter using a chromatin immunoprecipitation (ChIP) assay. 

**Figure 8:**
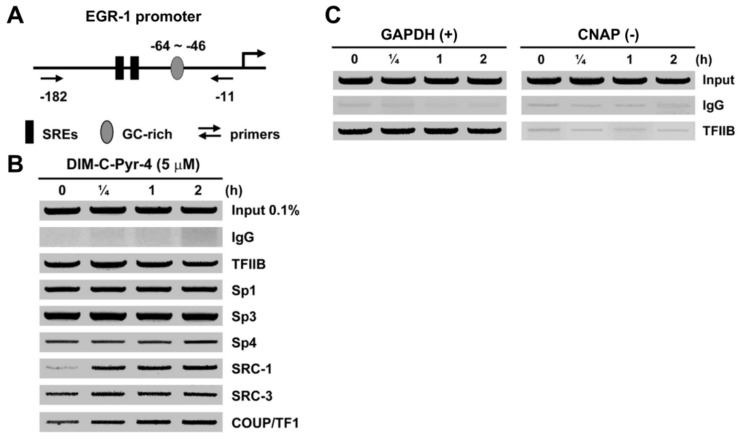
Chromatin immunoprecipitation assay. (**A**) Egr-1 promoter and positioning of PCR primers. (**B**) Chromatin immunoprecipitation (ChIP) assay of Egr-1 promoter. MCF-7 cells were treated with 5 μM DIM-C-Pyr-4 for 0, 0.25, 1.0, and 2.0 h, and cells were harvested and analyzed in a ChIP assay as described in the Materials and Methods section. (**C**) Binding of transcription factor II B (TFIIB) to the GAPDH promoter. The ChIP assay was also used to examine binding of TFIIB to the GAPDH promoter (positive control) and to exon 1 of centrosomal Nek2-associated protein 1(CNAP1) (negative control) as described in the Materials and Methods section.

Figure 8A schematically outlines the proximal region of the Egr-1 promoter and the primer positions. The results (Figure 8B) show that after treatment of MCF-7 cells with 5 μM DIM-C-Pyr-4 for 0.25–2.0 h, the band intensities associated with Sp1, Sp3, Sp4, and TFIIB were unchanged; COUP-TFI is constitutively bound to the promoter but the band intensity increased by 2- to 3-fold after treatment with DIM-C-Pyr-4. SRC-3 is constitutively bound to the Egr-1 promoter and was not affected by treatment with DIM-C-Pyr-4, whereas SRC-1 recruitment to the Egr-1 promoter was increased and ligand-dependent. The effects of DIM-C-Pyr-4 on the interactions of SRC-1 and SRC-3 with the Egr-1 promoter in the ChIP assay complement DIM-C-Pyr-4-induced interactions of VP-COUP-TFI with GAL4-SRC-1 but not GAL4-SRC-3 in a mammalian two-hybrid assay (Figure 1F). Figure 8B represents a positive control showing the binding of TFIIB to the GAPDH promoter but not exon 1 in the CNAP gene. These results demonstrate that DIM-C-Pyr-4 induces expression of Egr-1 in breast cancer cells through activation of a COUP-TFI/Sp transcription factor complex.

### 3.4. Molecular Modeling Studies

**Figure 9:**
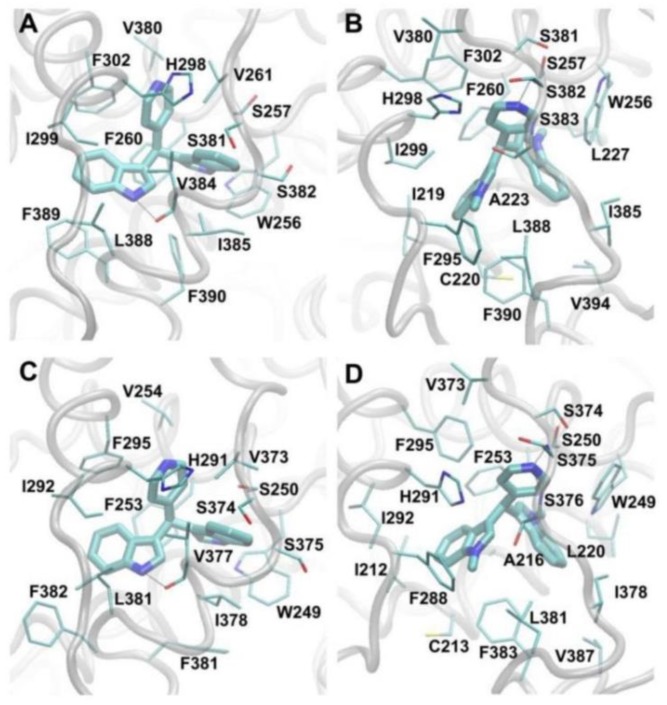
Molecular graphic images of (**A**,**C**) C-DIM-Pyr4 and (**B**,**D**) C-1,1-CH3-DIM-Pyr4 in complex with (**A**,**B**) COUP-TFI and (**C**,**D**) COUP-TFII. The compounds are shown in licorice representation. Interacting COUP-TFI/2 residues are shown in thin licorice representation, and COUP-TFI/2 backbone atoms are shown in transparent, gray tube representation. Hydrogen bonds are indicated using black, dotted lines.

In the studied simulations of DIM-Pyr4 in complex with COUP-TFI/II, C-DIM-Pyr4 forms non-polar interactions with V261/254 (COUP-TFI residue/COUP-TFII residue), H298/291, I299/292, V380/373, S382/375, V384/377, I385/378, L388/381, and F389/382, π–π interactions with the aromatic rings of W256/249, F260/253, and F302/295, as well as hydrogen bonds with the backbone carboxyl group of V384/377 and the side-chain hydroxyl group of S257/250. The interactions of key COUP-TFI/II residues to C-DIM-Pyr4 are presented in Figure 9A,C. In the studied simulations of C-1,1-CH3-DIM-Pyr4 in complex with COUP-TFI/II, C-1,1-CH3-DIM-Pyr4 forms non-polar interactions with I219/212, C220/213, A223/216, L227/220, F295/288, H298/291, I299/292, L388/381, F389/382, and V394/387, π–π interactions with the aromatic rings of W256/249 and F260/253, as well as hydrogen bonds with the backbone amide groups of S382/375 and S383/376. The interactions of key COUP-TFI/II residues to C-1,1-CH3-DIM-Pyr4 are presented in Figure 9B,D.

As both compounds occupy the same COUP-TFI/II binding pocket, the two compounds, for the most part, interact with the same COUP-TFI/II residues; the interactions between COUP-TFI/II residues and the two compounds are favorable. The average per-residue interaction free energy between the two compounds and COUP-TFI residues as well as COUP-TFII residues are decomposed into polar and non-polar contributions, independently, and selected interactions are presented in Supplementary Materials Figure S1. Both compounds interact strongly with residues W256/249, F260/253, H298/291, I299/292, and L388/381. Interestingly, despite both of the compounds occupying roughly the same COUP-TFI/II binding site, only C-DIM-Pyr4 interacts strongly with S257/250, V261/254, F302/295, V380/373, V384/377, I385/378, and F389/382, and only C-1,1-CH3-DIM-Pyr4 interacts strongly with I219/212, C220/213, A223/216, L227/220, F295/288, S383/376, S381/374, S382/375, F383, and V394/387.

Of the aforementioned residues, previous mutagenesis experiments show that mutations to W249, S250, F253, and V254 significantly decrease the activity of COUP-TFII [39]. Our modeled structure of the C-DIM-Pyr4:COUP-TFII complex is in accordance to these mutagenesis studies [39], with all four COUP-TFII residues interacting strongly with the active C-DIM-Pyr4. At the same time, C-1,1-CH3-DIM-Pyr4 strongly interacts with W249 and F253 but not with S250 and V254. Since both C-DIM-Pyr4 and C-1,1-CH3-DIM-Pyr4 are predicted to interact strongly with W249 and F253, it is possible that these residues are critical for compound binding to—and thus may also affect the activity of—COUP-TFII. Additionally, since only C-DIM-Pyr4 is predicted to interact strongly to S250 and V254, it is possible that these residues may have a critical role in COUP-TFII activity. Similarly, given the predicted highly similar binding of the two compounds to COUP-TFI in comparison to COUP-TFII, it could be hypothesized that the above may also hold for the COUP-TFI residues W256, S257, F260, and V261 which correspond to COUP-TFII residues W249, S250, F253, and V254, respectively.

Visual inspection and root mean square deviation (RMSD) comparing the final stimulation overall structure (extracted at 30 ns) of COUP-TFII in complex with C-DIM-Pyr4 and COUP-TFII in complex with C-1,1-CH3-DIM-Pyr4 to the X-ray structure of the unbound COUP-TFII [39] show that the structures are very similar. The RMSD of COUP-TFII in structure of the un-complexed COUP-TFII [39] is 2.5 Å and 1.5 Å, respectively, which originate primarily from the binding pocket (COUP-TFII α3-helix residues 212–220, α5-helix residues 249–257, α7-helix residues 291–298, α10-helix residues 378–383, and AF2-helix residues 392–394). The RMSD of the interacting domains of COUP-TFII in complex with C-DIM-Pyr4 and COUP-TFII in complex with C-1,1-CH3-DIM-Pyr4 compared to the X-ray structure of the un-complexed COUP-TFII [39], respectively, is 1.9 Å and 1.0 Å for α3-helix residues 212–220, 1.2 Å and 0.8 Å for α5-helix residues 249–257, 2.9 Å and 0.7 Å for α7-helix residues 291–298, 5.1 Å and 3.4 Å for α10-helix residues 378–383, and 3.9 Å and 1.3 Å for AF2-helix residues 392–394. Overall, the structure of the modeled bound COUP-TFII are more similar to the X-ray structure of the un-complexed COUP-TFII [39] in three of the helical domains (α3-helix residues 210–220, α5-helix residues 249–257, and α7-helix residues 291–298) than in the other two of the helical domains (α10-helix residues 378–383 and AF2-helix residues 392–394). For the former three helical domains (i.e., α3-helix residues 210–220, α5-helix residues 249–257, and α7-helix residues 291–298), slight displacement of the three helical domains is observed for COUP-TFII binding to C-DIM-Pyr4 allowing the receptor to embrace/accommodate the agonist: in the structure of COUP-TFII binding to C-1,1-CH3-DIM-Pyr4, the conformation of the three helical domains is nearly identical to that of the X-ray structure of the un-complexed COUP-TFII [39]. The main differences in the binding pocket of the two bound structures compared to the un-complexed X-ray structure of COUP-TFII (39) originate from the latter two helical domains (i.e., α10-helix residues 378–383 and AF2-helix residues 392–394). In the X-ray structure of the un-complexed COUP-TFII [39], the absence of a bound compound allows the α10-helix to be more proximal to the α3-helix. In the structures of COUP-TFII in complex with both C-DIM-Pyr4 and C-1,1-CH3-DIM-Pyr4, the positioning of the α10-helix is adjusted to accommodate the compounds in the binding pocket between the α10-helix and α3-helix. In our future studies we plan both receptor–ligand binding and transactivation studies using wild-type and mutant COUP-TFI constructs to complement the modeling results. 

## 4. Discussion

COUP-TFI is an orphan receptor and a member of the nuclear receptor family of transcription factors. COUP-TFI contains the typical domain structure of nuclear receptors, and like many other orphan receptors for which endogenous ligands have not been identified, mouse transgenic studies have identified important tissue-specific functions for this protein [60,61,62,63,64]. For example, COUP-TFs are differentially expressed in the embryo, and in COUP-TFI knockout animals, most of the animals die perinatally and only a small percentage (1–2%) survive up to 3–4 weeks after birth. Failure to express COUP-TFI results in pronounced neuronal deficits, increased demyelination in the central nervous system, and delayed axon myelination [56]. COUP-TFI also participates in regulation of multiple genes through direct or indirect interactions with cis-elements and/or other transcription factors [8,10,24]. 

In this study, we screened a series of C-DIM compounds as possible COUP-TFI agonists since previous studies in this laboratory have identified 1,1-bis(3′-indolyl)-1-(p-substituted phenyl) methane analogs as agonists for orphan receptors PPARγ and Nur77 [35,36,37,38]. Compounds containing para-phenyl (DIM-C-pPhC6H5) and para-t-butyl (DIM-C-pPhtBu) substituents preferentially activate PPARγ [35,36]; para-methoxy (DIM-C-pPhOCH3) and an unsubstituted analog (DIM-C-Ph) activate Nur77 [38]; and the para-trifluoromethyl compound (DIM-C-pPhCF3) activates both receptors. These C-DIM compounds did not activate GAL4-COUP-TFI (data not shown). We also screened a series of heteroaromatic C-DIMs, and as illustrated in Figure 1, DIM-C-Pyr-4 activated GAL4-COUP-TFI. Induction of luciferase activity by the DIM-C-pyridyl analogs was highly regiospecific with respect to location of the pyridine nitrogen; DIM-C-Pyr-4 was active, whereas the meta-substituted compound (DIM-D-Pyr-3) was completely inactive and the ortho-pyridyl compound only exhibited minimal activity (Figure 1C). Most other heteroaromatic C-DIM analogs, including those with thiophenyl, furanyl, pyrrole, and indolyl groups, were inactive (Figure 1C). Moreover, methyl substitution on the indole ring (1,1- and 2,2-dimethyl DIM-C-Pyr-4) and the DIM-C-Pyr-4 N-oxide derivative was also inactive and methyl substitution (5,5′-dimethyl) on the phenyl moiety of the indole ring resulted in decreased activation of GAL4-COUP-TFI in breast cancer cell lines. These results demonstrate that among the heteroaromatic C-DIM analogs DIM-C-Pyr-4 stands out as a unique activator of COUP-TFI in breast cancer cells, and similar results have been obtained in the LNCaP prostate cancer cell line (unpublished data). 

COUP-TF1 has not been extensively investigated for its functions in cancer; however, a recent study [64] reported that COUP-TFI has some prognostic significance in dormant disseminated cells in breast cancer patients. However, reports that COUP-TFI both activates and inhibits estrogen signaling in breast cancer cells [65,66] suggests that applications of compounds such as DIM-C-Pyr4 for treating breast and other cancer requires further investigation.

COUP-TFI-mediated transactivation is enhanced by interactions with several nuclear factors including SRC-1 and SRC-2 which have been extensively characterized as “prototypical” coactivators of steroid hormone receptors and other nuclear receptors [67,68,69,70,71,72]. For example, SRC-1 and SRC-2 potentiate COUP-TF-dependent activation of a CYP7A-DR4 derived construct in HeLa cells [29] and SRC-1 enhances COUP-TFI/Sp1-mediated upregulation of Egr-1 in rUGM cells [27]. Results in Figure 1F show that in mammalian two-hybrid assays in MCF-7 cells, DIM-C-Pyr-4 induced transactivation in cells transfected with VP-COUP-TFI and GAL4 chimeras with SRC-1 and SRC-2, whereas these interactions were not induced or minimally induced in cells transfected with chimeras expressing other coactivators (SRC-3, TRAP220, CARM-1, and PGC-1) or the corepressor SMRT. These results demonstrate that the structure-dependent activation of GAL4-COUP-TFI by DIM-C-Pyr-4 and no other heteroaromatics is complemented by the specificity of DIM-C-Pyr-4-induced coactivator-COUP-TFI interactions in MCF-7 cells. We are planning to further investigate ligand- and kinase-dependent interactions of COUP-TFI with coactivators and other nuclear cofactors and their effects on multiple COUP-TF1-regulated genes.

Activation of steroid hormone receptors and orphan receptors by endogenous or exogenous compounds is complex and dependent not only on receptor–agonist interactions but also kinase-dependent activation (phosphorylation) of these receptors and coactivators [53,54,55,56,57]. Figure 2 illustrates that DIM-C-Pyr-4 activated GAL4 chimeras containing both the N- and C-terminal domains of COUP-TFI and the structure-dependent activation of these chimeras by C-DIM compounds was similar to that observed for GAL4-COUP-TFI (Figure 1). These results suggest that activation of COUP-TFI by DIM-C-Pyr-4 may also be associated with kinase activation since the N-terminal domain of COUP-TFI does not contain the ligand binding domain. Among the several kinase inhibitors that were investigated, significant inhibition of GAL4-COUP-TFI and GAL4-COUP-TFI-ΔC activation by DIM-C-Pyr-4 was observed with PI3-K and cAMP/PKA inhibitors, whereas only the PI3-K inhibitors blocked activation of GAL4-COUP-TFI-ΔN. Thus, DIM-C-Pyr-4 differentially activated the N- and C-terminal domains of COUP-TFI, suggesting that the effects of DIM-C-Pyr-4 are complex and include activation of kinases.

Kinase-dependent activation of COUP-TFI by DIM-C-Pyr-4 does not necessarily exclude a role for this compound as a COUP-TFI agonist since other nuclear receptors such as ERα also activate kinases and exhibit receptor agonist activities [53,54,55,56,57]. Therefore, based on the inhibitory effects of PI3-K inhibitors on DIM-C-Pyr-4-mediated activation of GAL4-COUP-TFI (Figure 3A), we confirmed that DIM-C-Pyr-4 induced PI3-K-dependent phosphorylation of Akt in both MCF-7 and ZR-75 cells (Figure 4A). However, we also observed that DIM-C-Pyr-3 induced PI3-K-dependent phosphorylation of Akt and this compound does not activate GAL4-COUP-TFI (Figure 1) indicating that activation of this kinase did not necessarily lead to activation of GAL4-COUP-TFI. We further investigated the COUP-TFI agonist activity of DIM-C-Pyr-4 in mammalian two-hybrid assay in MCF-7 cells where it was shown that DIM-C-Pyr-4 but no other heteroaromatic C-DIMs induced COUP-TFI-SRC-1/SRC-2 interactions (Figure 1F). Moreover, results in Figure 4B,C show that DIM-C-Pyr-4, but not the inactive DIM-C-Pyr-3 or DIM-C-Pyr-2 isomers, induced SRC-1 interactions with COUP-TFI-ΔN (containing the ligand binding domain), and the interactions induced by DIM-C-Pyr-4 were not significantly blocked by PI3-K inhibitors. These data suggest that DIM-C-Pyr-4 can also act, in part, as a COUP-TFI agonist, and current studies are focused on synthesizing suitable fluorescent/radioactive analogs to more closely examine interactions of DIM-C-Pyr-4 with the ligand binding domain of COUP-TFI.

COUP-TFI activates Egr-1 in rUGM cells and this involves COUP-TFI/Sp interactions with the proximal GC-rich region of the Egr-1 promoter [27]. This mechanism is observed for the induction/inhibition of other genes by COUP-TFI [8,10,11,12,13,14,15,16,17,18,19,20,21,22,23,24] and several other nuclear receptors [68]. Figure 5 and Figure 6 demonstrate that DIM-C-Pyr-4 also induced Egr-1 mRNA in MCF-7 and ZR-75 cells, and RNA interference showed that this response was inhibited by COUP-TFI and also iSp1 and iSp3 where Sp3 was the major Sp protein involved in COUP-TFI/Sp3-mediated activation of Egr-1. The effects of these small inhibitory RNAs on COUP-TFI and Sp proteins and their inhibitory effects on induction of Egr-1 protein by DIM-C-Pyr-4 were observed (Figure 7). Results of the ChIP assay (Figure 8) demonstrate constitutive binding of Sp proteins to the Egr-1 promoter, and this is consistent with results of similar studies on other GC-rich promoters in breast cancer cells [58,59]. The DIM-C-Pyr-4-dependent recruitment of COUP-TFI and SRC-1 to the Egr-1 promoter is consistent with results of the mammalian two-hybrid assay (Figure 1F) and the reported coactivation of COUP-TFI-mediated transactivation by SRC-1 [27,28,29]. 

The two compounds investigated in the modeling and simulation studies, DIM-C-Pyr4 and 1,1-CH3-DIM-Pyr4, are nearly identical, except that the latter compound contains N-methyl groups instead of hydrogen atoms as in DIM-CPyr4. Despite their similar chemical structures, our computational studies suggest that their subtle difference results in somewhat different binding modes in complex with COUP-TFI/II. Our results suggest that this is due to the fact that the methyl groups of 1,1-CH3-DIM-Pyr4 appear to add bulk to the compound and impede its ability to form hydrogen bonding with the backbone carboxyl group of V384/377, which occurs between DIM-C-Pyr4 and COUP-TFI/II. Instead, in 1,1-CH3-DIM-Pyr4, the methyl groups are attracted to COUP-TFI/II residue motif 219/212–227/220. Due to the somewhat different binding modes of the two compounds, only 1,1-CH3-DIM-Pyr4 (inactive in transactivation) interacts strongly with COUP-TFI/II residues I219/212, C220/213, A223/216, L227/220, F295/288, S383/376, S381/374, S382/375, F383, and V394/387; while only DIM-C-Pyr4 interacts strongly with COUP-TFI/II residues S257/250, V261/254, F302/295, V380/373, V384/377, I385/378, and F389/382 and these differences may influence their different activities as activators of COUP-TFI. Of the aforementioned residues, previous mutagenesis experiments show that mutations to W249, S250, F253, and V254 significantly decrease the activity of COUP-TFII [46]. Our modeled structure within the simulation of the DIM-C-Pyr4:COUP-TFII complex is in accordance to these mutagenesis studies [46], with all four COUP-TFII residues interacting strongly with the active DIM-C-Pyr4. At the same time, 1,1-CH3-DIM-Pyr4 forms strong interactions with W249 and F253 but not S250 and V254. Since both DIM-C-Pyr4 and 1,1-CH3-DIM-Pyr4 are predicted to interact strongly with W249 and F253, it is possible that these residues are critical for compound binding to—and thus may also affect the activity of—COUP-TFII. Additionally, since only DIM-C-Pyr4 is predicted to interact strongly to S250 and V254, it is possible that these residues may have a critical role in COUP-TFII activity. Similarly, given the predicted highly similar binding of the two compounds to COUP-TFI in comparison to COUP-TFII, it could be hypothesized that the above may also hold for the COUP-TFI residues W256, S257, F260, and V261 which correspond to COUP-TFII residues W249, S250, F253 and V254, respectively. These results demonstrate for the first time that COUP-TFI can be activated by an exogenous compound, and in breast cancer cells which express COUP-TFI, this results in induction of Egr-1.

## Supplementary Materials

The following are available online https://www.mdpi.com/2073-4409/8/3/220/s1, Figure S1: Average interaction free energies (kcal/mol) for (A) COUP-TFI and (B) COUP-TFII residues in complex with DIM-C-Pyr4 and 1,1-CH3-DIM-Pyr4.
