# Supplementary material for: Activation of COUP-TFI by a Novel Diindolylmethane Derivative
Source: Cells. 2019 Mar 7;8(3):220. doi: 10.3390/cells8030220 (PMC6468570; doi:10.3390/cells8030220)
Supplement: Supplementary file 1 [file cells-08-00220-s001.pdf]

Supplementary Materials

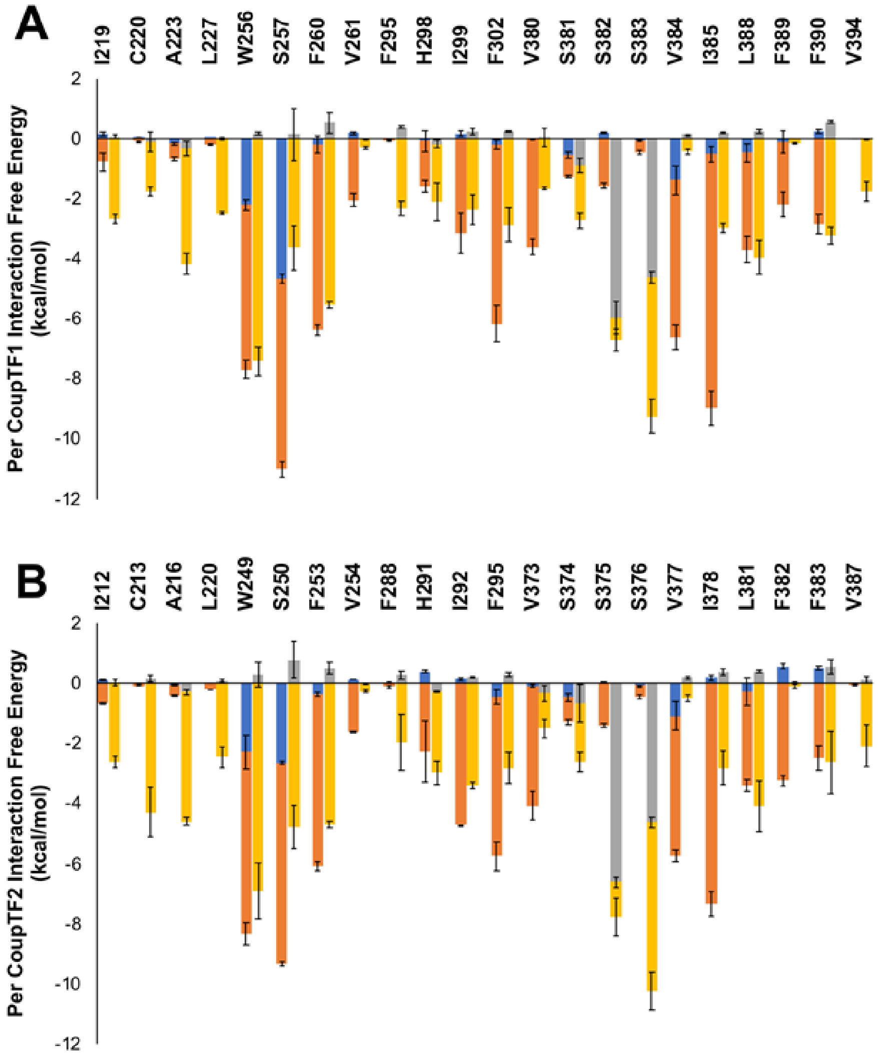

Figure S1. Average interaction free energies (kcal/mol) for (A) COUP-TFI and (B) COUP-TFII residues in complex with DIM-C-Pyr4 (first bar per residue) and 1,1-CH_3-DIM-Pyr4 (second bar per residue). The interaction free energies are decomposed into polar (blue or grey) and nonpolar (orange or yellow) contributions. The sum of polar and nonpolar contributions corresponds to the total average per COUP-TFI/II residue interaction free energy. Only residues with average interaction free energies less than -1.0 kcal/mol in either the DIM-C-Pyr4:COUP-TFI/II or 1,1-CH_3-DIM-Pyr4:COUP-TFI/II complexes are presented. Results were calculated using the ensemble of snapshots extracted from simulation trajectories of the most energetically favored binding conformations. The average and standard deviation values for the polar and nonpolar components of the interaction free energies were calculated through block averaging the final 20 ns of each of the 30 ns MD simulations into four segments of 5 ns each.
